# Maritime Quarantine1Read before the Alumni Association, Niagara University Medical College, May 9, 1894.

**Published:** 1894-06

**Authors:** William A. Wheeler

**Affiliations:** M. D., U. S. M. H. Surgeon; Ellis Island, N. Y.


### MARITIME QUARANTINE.[1]

By WM. A. WHEELER, M. D., U. S. M. H. Surgeon.

I am deeply conscious of the distinction which an invitation to appear here has conferred upon me, and doubly pleased to be with you because of the personal interest I must always feel in the growth and prosperity of your Alma Mater, a feeling natural to one who was in at the birth, so to speak, as Virgil puts it, "all of which I saw and part of which I was." Your chairman has asked me to make a contribution to this annual symposium of yours, and while I may be surprised at his judgment, you must in no way relieve him from responsibility as to the character of entertainment he provides.

It is of Maritime Quarantine in this Country that I propose to speak for a few moments today, not that I pretend to know so much about the subject, but because, from what I do know, I have derived some very positive opinions, which may or may not be correct, but which, at least, it will relieve my mind to express here before you. It is of no use for me to tell you that the word quarantine does not mean today what it once did. Sanitary inspection is too long, and we will continue to use the word suggestive of the Lenten season. This subject has been brought very forcibly to our minds and, indeed, to all thinking minds, the last two years, and out of the prominence given the subject some good to the public generally ought to come. The public, always ready to criticize, as is right, especially that part of it which gives voice to its

1. Read before the Alumni Association, Niagara University Medical College, May 9, 1894.

sentiments through the columns of the press, is asking the question: Are we abreast of the times in this matter, or do we not cling to methods which belong rather to the dark ages? And England is quoted as a country which has discarded quarantine as unsuited to modern needs.

Before we condemn our system, let us first explain what is meant at the present time by quarantine, and what it is designed to accomplish. It does not imply necessarily a detention, but simply a careful examination of persons and their belongings, to the end that disease may not be brought to us from abroad. There are two methods of fighting epidemic disease—one by preventing, if possible, its entrance to your home; the other by keeping your house so clean and well ordered that it will not stay with you when it comes. We are trying the former plan; England, the latter. She prefers to try and keep her house clean, having a small establishment and perfect control of every part of it; and, depending for her prosperity, as she does, so largely upon commerce, she is unwilling to impose any restrictions upon it. We, on the other hand, have such a large house, or, indeed, so many of them, inhabited by such a variety of tenants, who control their own apartments, that we distrust our ability to keep clean and so bar the gates that disease may not get a foothold. Consider for a moment the extent of our territory and the ease and speed with which communication is kept up between all sections. We have a land three thousand miles in width and two thousand miles deep, if I may be allowed the expression, with a great variety of climate. It is possible for an army of people to arrive in one of our Atlantic ports today, and tomorrow be scattered in fifteen different states. It is a common thing for a thousand people to start out by special train from New York, bound for the West, reaching Chicago in thirty-six hours, and diverging from there in all directions. Can we afford to allow these people, fresh from a crowded ship, which has been their home for the last ten or fifteen days, scatter all over these fifty states and territories without knowing or caring if they are free from disease? Can we keep track of these thousands and watch them in their wanderings throughout this vast country? Now, look at the condition in England. She has, together with Scotland, an area about equal to that of Indiana and Illinois, with a cool temperate climate, all her people under one sanitary system, which is the growth of centuries and the admiration of those familiar with it. She has no immigration, as we understand it, and her commerce is her very life and must be fostered. The strangers who enter her gates are merely breaking their journey to other lands, and their movements can be and are accurately known. They are passing through by the shortest and quickest routes, often not remaining over night on English soil. She does not need the careful guarding of her gates as with us, and we could not adopt her close and efficient system of sanitary espionage if we would. Commerce seeks us of its own accord, and we feel justified in imposing such slight restrictions as we believe needed for our protection.

In view of the totally different conditions existing in the two countries, and also viewed in the light of experience, it would seem that both countries were wise in their methods. We have a coast line many thousands of miles in extent, included in the domain of twenty-one different states. Along this coast line are found scores of ports through which the foreign commerce of the country is conducted, and it is at these gates that guards, more or less efficient, have been placed. In some cases these quarantine establishments or plants are maintained by the federal government, with the consent of the states in which they are located, and this is especially the case where the revenues would amount to very little. In most instances they are maintained by the states in which the ports are situated, and in some cases the port itself keeps up its own quarantine. In our larger ports the revenues derived from the dues or tax imposed upon shipping is more than sufficient to defray all expenses of maintenance, but in the cases of the smaller ports an annual appropriation must be made by the state or city, and the federal quarantines are directly provided for by Congress. The men in control of these quarantines are federal, state or city officers, as the case may be, all independent of each other, and working on separate lines. Until one year ago they were absolutely free from any supervision save by state or municipal officers, with the result that while some were carefully and honestly administered, and therefore trustworthy, others were quite the reverse. When the expense of maintaining an efficient quarantine must be met by an annual appropriation from a small and impoverished community, there naturally exists a great temptation to curtail its efficiency, and the country at large may be the sufferer. When the income from fees imposed upon shipping largely exceeds the cost of maintaining a first-class quarantine, we have a right to expect the latter, and I am bound to say such is usually the case; but there is a strong temptation to make use of the revenues thus derived to serve political rather than sanitary ends.

An effective quarantine plant today consists of an inspection or boarding station, at which vessels are stopped and their people examined, and a station of refuge, to which the sick are removed, and all others whom it is necessary to detain, together with their baggage. In many instances these two stations can be very well combined. The diseases for which, by common consent, a sanitary inspection is made, are: smallpox, typhus, cholera and yellow fever, and provision must be made for sterilizing all personal effects, which involves the erection of buildings and expensive apparatus. Besides hospital buildings, houses for the detention of the well until free from danger must be constructed, and wharves or lighters with tugs maintained. Thus you see a very considerable expense is needed for construction alone, to say nothing of maintenance.

Now, why should seaports, or states in which the seaports are situated, be put to this expense when the benefit is for the whole country at large? And, conversely, why should the people of the whole country be content to leave it with the sea coast states and cities to provide such protection as they may see fit? Maritime quarantine deals only with foreign commerce, and the regulation of the latter is by the constitution vested in the federal government. The general government regulates the imposition and collection of all duties; it imposes a tax upon all foreign tonnage; it prescribes the conditions under which, and the manner in which, passengers may be brought to our shores; it imposes a head tax upon foreigners for the purpose of defraying the expenses incident to carrying out its own laws; it goes to the extent of compelling foreign vessels to take on pilots when entering our ports, or, at least, pay pilot dues. It has built a chain of custom houses from Eastport to Brownsville on the Atlantic, and from Port Townsend to San Diego on the Pacific, each with its detectives to see that not a diamond or silk dress gains free admission to this country, assisted by a navy of some twenty-five vessels, all fully officered and manned to prevent the possible smuggling of a box of cigars or a seal skin, and yet it leaves to the State of Florida or Georgia the task of keeping out such a frightful disease as yellow fever, and to New York the duty of repelling cholera. The strange spectacle is presented at our largest seaports, of United States officers waiting for the inspection and release of all foreign vessels by state or municipal officers, before going on board.

I am not ignorant of the dangers which may arise from too great centralization of power in the hands of our National Government, and I am a great believer in the doctrine of allowing each state to mind its own affairs whenever that does not interfere with the affairs of its neighbors; but we have here an affair which is of vital interest to every one of the states of the Union. It is the whole country which is building up the commerce of Boston, New York, Philadelphia and Baltimore, and a very small percentage of their trade is furnished by the people of their respective states. The general government, at these and all seaports, allows of no state interference with its custom and immigration inspection. Why should it make an exception of the sanitary inspection? The whole country is aroused to the needs of our sea coast defenses against a foreign foe, and no states are so loud in their demands upon the general government for liberal appropriations for this end as those states in which the defenses will be located. No states are so persistent in their demands for an increased naval force to protect their commerce as these same seaboard states; but when a revenue is to be derived, as is the case in the quarantines of our larger ports, not only is the government not called upon to provide defenses against disease, but it is requested not to interfere in the matter as regulated by the states. One of the functions of government is to provide protection to the governed against a foreign foe, and we can certainly claim that two, at least, of these contagious diseases are foreign. Cholera and yellow fever are not native, nor have they acquired any rights by reason of long residence.

You are aware that the New York Academy of Medicine has prepared and had offered in Congress a bill establishing a Bureau of Public Health in the Interior Department. This bill does not change practically the status of the maritime quarantines, but leaves them in the control of their respective state or municipal officers, subject only to supervision by the General Government, as is the case now, which supervision is now and will continue to be more or less unsatisfactory and a constant source of irritation, galling to the state officers, who are accountable only to their respective states, and most disagreeable to the federal officers, to whom is allotted the uncongenial labor of supervising the methods and sanitary systems of quarantine officers who are not connected with the general government. I consider the present system weak, in that the responsibility is a divided one; instead of being a case of two heads better than one, it is more likely to prove a case of the proverbial broth which was spoiled by too much supervision. To insure a complete system of maritime quarantine, uniform in its methods whether applied at the Atlantic or Pacific or in the Gulf, uniform in its equipment regardless of the revenues of any given port, affording as much protection as is possible in any quarantine, the work must be done by the general government. I am not opposed to the creation of a Bureau of Public Health at the national capital, and it may very properly be attached to the Department of the Interior, and have control in general of all matters affecting the sanitary welfare of our people, though I can foresee the inevitable conflict between state and national authority, which is bound to arise if such a bureau is to have executive powers. Whether it is best, all things considered, for the general government to take away from the several states the responsibility of caring for the health of their own citizens, with all that that implies, is surely a debatable question, but that it belongs to the government to afford us all of the protection possible against the invasion of disease from abroad seems to me to admit of no argument. Our treasury department regulates and controls the importation of all merchandise and peoples into these United States, and to it should be given the absolute control of our sea coast quarantines. If a bureau already exists in the treasury department, familiar with this work and fully capable of conducting it, it is the part of common sense to make use of it for this purpose. If such a bureau exists and it has not fully demonstrated its ability to manage an efficient system of coast quarantines, then let a new bureau be established for this special work. For a number of years the treasury department has maintained in the Gulf of Mexico and on the South Atlantic coast refuge stations, to which state quarantine officers in the vicinity have sent vessels needing purification. These have been built because the Southern States, most of them, have been too poor to provide such facilities, and the revenue would in no way suffice for their maintenance after being built, and the whole South is annually exposed to the introduction of yellow fever from the West Indies. The general government, in addition to these refuge stations, has established, at the entrance to the Delaware and Chesapeake bays, inspection stations for the purpose of examining all vessels coming into these waters, and it is altogether an unnecessary burden upon commerce that the seaports in these bays should require an additional sanitary inspection. On the Pacific coast, the national government has already established both inspection and refuge stations for San Francisco, San Diego and Puget Sound. These are fully equipped and thoroughly prepared for any possible demand that may be made upon them.

The treasury department last year, in view of the widespread belief that cholera would again appear in Europe, was enabled with its own officers to establish a quarantine inspection at twelve foreign ports, from which the disease was most likely to reach us. What protection this afforded us I will not attempt to discuss, but it could not have been attempted by any other than the general government. It thus appears that there exists already the nucleus of a federal quarantine, which could readily be developed and extended to afford to all the people of all the states the maximum security possible in any quarantine, with the minimum of delay and expense to commerce under the charge of men specially trained for the work, making of it a profession for life, independent of state influences or party politics, working with a single purpose and for a single object and all responsible to a common head. We have but to look across our northern border to find the condition such as I speak of, the Canadian government having established most admirable quarantines at her principal ports and not leaving to the separate provinces the task of providing defenses for the whole Dominion.

Ellis Island, N. Y.

